# A 10-year population-based nationwide descriptive analysis of pediatric emergency care

**DOI:** 10.1186/1471-2431-14-100

**Published:** 2014-04-10

**Authors:** Mei-Jy Jeng, Yu-Sheng Lee, Pei-Chen Tsao, Chia-Feng Yang, Yu-Cheng Luo, Wen-Jue Soong

**Affiliations:** 1 Institute of Emergency and Critical Care Medicine, National Yang-Ming University, Taipei, Taiwan; 2 Department of Pediatrics, School of Medicine, National Yang-Ming University, Taipei, Taiwan; 3 Department of Pediatrics, Taipei Veterans General Hospital, 201, Section 2, Shih-Pai Road, Taipei 11217, Taiwan

**Keywords:** Children, Emergency, Hospitalization, National health insurance research database

## Abstract

**Background:**

Pediatric emergency care medicine is an important field of health care. This study aimed to investigate the 10-year pediatric emergency care in children aged 0-17 years old in Taiwan.

**Methods:**

Systematic random samples from the National Health Insurance Research Database of Taiwan in the period 2000-2009 were analyzed. Children recorded as undergoing emergency care were enrolled and divided into different age groups. The frequency of emergency visits, age, cost per visit, seasonality, number of hospitalizations, and diagnosis were analyzed.

**Results:**

A total of 764,598 children were enrolled. These children accounted for 25% of all emergency cases and their mean age was 6.1 years. Children aged 0-5 years formed the largest group, with male predominance (57.5%). The incidence of emergency visits was 29133 ± 3104 per 100,000 children per year (mean ± SD). Acute upper airway infection, fever, and acute gastrointestinal illness were the most common diagnoses among all non-hospitalized children. Some (4.51%) required subsequent hospitalization and their most common diagnoses were fluid/electrolyte disorder, upper/lower airway infection, and acute gastrointestinal illness. The group of children aged 12-17 years had cases of traumatic injury and childbirth.

**Conclusions:**

In Taiwan, 25% of individuals seeking emergency care are children, mostly aged 0-5 years old. Costs and disease patterns vary among different age groups. Preventive measures targeting all children should focus on respiratory and gastrointestinal diseases, but should target different diseases for different age groups to improve child health.

## Background

Emergency care medicine is a very important field of health care. Children are not small-sized adults and there are many differences in the physical condition and daily activities between adults and children. Like in the United States, the most frequent cause of death among children in Taiwan is unintentional injury [1,2]. Acute illnesses are also a common reason for children to seek emergency care. Thus, a comprehensive study of pediatric emergency care is an important way of improving the quality of pediatric medical care.

There were around 22.27-23.12 million people living in Taiwan over the period of 2000-2009 (mean, 22.73 ± 0.28 million), including 4.74-5.78 million people (mean, 5.27 ± 0.34 million) younger than 18 years old (National Statistics, Taiwan, R.O.C.) (Table 1) [3,4]. As such, children aged 0-17 years old account for approximately one-fourth to one-fifth of the general population. Still, there is a lack of comprehensive reports exploring pediatric emergency care covering the last 10 years.

**Table 1:** National household population, national health insurance (NHI) coverage, and incidence of children requiring emergency care in Taiwan during the period 2000-2009

**Age/Year**	**2000**	**2001**	**2002**	**2003**	**2004**	**2005**	**2006**	**2007**	**2008**	**2009**	**Mean ± SD**
***All age***											
Population (millions)	22.27	22.41	22.52	22.60	22.69	22.77	22.88	22.96	23,04	23.12	22.73 ± 0.28
NHI covered (millions)	21.40	21.65	21.87	21.98	22.13	22.31	22.48	22.80	22.92	23.03	22.26 ± 0.55
Coverage ratio (%)	96.1	96.6	97.1	97.3	97.6	98.0	98.3	99.3	99.5	99.6	97.9 ± 1.2
***0-17 years old***											
All population	5,779,069	5,662,521	5,544,533	5,429,950	5,345,047	5,242,928	5,107,181	5,002,123	4,868,304	4,745,159	5,272,682 ± 342,467
5% population*	288,953	283,126	277,227	271,498	267,252	262,146	255,359	250,106	243,415	237,258	263,634 ± 17,123
5% ER visits*	75,912	73,850	82,515	69,057	79,794	79,278	73,360	73,347	71,056	86,429	76,460 ± 5,432
5% Hospitalized*	1,112	1,800	2,240	1,882	3,719	3,678	3,135	3,347	3,606	3,429	2,795 ± 947
5% Non- hospitalized*	74,800	72,050	80,275	67,175	76,075	75,600	70,225	70,000	67,450	83,000	73,665 ± 5,274
ER visits (/100,000/year)	26,271	26,084	29,764	25,436	29,857	30,242	28,728	29,326	29,191	36,428	29,133 ± 3,104
Hospitalized	385	636	808	693	1,392	1,403	1,228	1,338	1,481	1,445	1,081 ± 407
Non- hospitalized	25,887	25,448	28,956	24,742	28,466	28,839	27,500	27,988	27,710	34,983	28,052 ± 2,838
ER visits (/1,000/month)	22	22	25	21	25	25	24	24	24	30	24 3

*Data are presented as 5% of original whole value.

The National Health Insurance (NHI) program, providing comprehensive medical care to all residents, was started in Taiwan in 1995. Its population coverage was 96.1% in 2000 and gradually increased to 99.6% in 2009 (Table 1) [4,5]. The database of the NHI program can reliably represent medical phenomena affecting all individuals living in Taiwan, including children. Various researches on children-related diseases using the National Health Insurance Research Database (NHIRD) [6-26] or targeting emergency care [27-30] have been published. However, there is a paucity of reports on the comprehensive and descriptive analysis of pediatric emergency care.

Subsequent hospitalizations following emergency care are important because these imply more severe cases. A published report on the epidemiology of emergency care in Taiwan from 2000 to 2004 covers all ages [27] but does not analyze subsequent hospitalizations. A thorough investigation that includes hospitalizations is crucial for understanding current pediatric emergency care. Long-term clinical data reflecting the true state of the patients will be helpful in making health policy changes, improving health care quality, and designing new medical or policy interventions.

The purpose of this study was to analyze the epidemiology, disease patterns, and subsequent hospitalizations of children younger than 18 years old who required emergency care in the past 10 years.

## Methods

### Data sources

Systematic sampling datasets from Taiwan’s National Health Insurance Research Database (NHIRD) from 2000 to 2009 were used for the computerized analysis. The systematic sampling data claims were released officially from the Bureau of NHI (BNHI) of Taiwan for academic use, including random samples of 0.2% of the ambulatory care expenditure by visits and 5% of the in-patient expenditure by admission extracted by a systematic sampling method on a monthly basis. Thus, the datasets were representative of the whole population of Taiwan who sought medical help in the period of 2000-2009. There had been published scientific reports based on these systematic sampling datasets [27,31].

These datasets contained information that included patient’s age, sex, admission date, discharge date, diagnosis, medical expenses, medication expenses, laboratory examination items, and operational codes. These datasets, provided by the NHIRD, consisted of aggregated secondary data without personal identification. The Institutional Review Board of Taipei Veterans General Hospital approved the study (VGHIRB No. 2012-06-006A).

### Data analysis

Information on children younger than 18 years old who were recorded as having an emergency visit, which was defined as having a record of an emergency diagnostic fee being charged, were collected. If there was a code (PART_NO = 903) that represented an infant who was younger than 2 months old and attached to parental health insurance, the individual was recognized as an infant. If data was present indicating any inpatient expenditure (5% of originally whole data) and there was a record of a subsequent hospitalization, these children were classified as a child that had undergone hospitalization. If data was present on the ambulatory care expenditure without any record of subsequent hospitalization, these children were classified as non-hospitalized and values from the 0.2% ambulatory systematic sampling data were multiplied by 25 to represent the same percentage as the in-patient dataset covering 5% of the population.

However, because Taiwan’s NHIRD for emergency visits could not simply be combined with the hospitalization records of the years between 2000 and 2003, the case number was likely to be underestimated during the earlier time period. As such, the hospitalized case numbers were also calculated specifically for the period of 2004 to 2009 in addition to the period of 2000 to 2009, because the medical providers were strictly requested to report patients’ emergency fee together with their subsequent hospitalization fee to BNHI during this time.

The enrolled children were divided into three age groups. The first was 0-5 years old group, which was sub-grouped into the <12 month-old infant group and the 1-4 year old group. The second and third groups were 6-11 and 12-17 year-old groups, respectively. Their frequency of emergency visits, seasonality, diagnosis, and cost per visit were analyzed and compared. In the analysis of the seasonal distribution, spring consisted of February, March, and April; summer consisted of June, July, and August; autumn consisted of September, October, and November; and winter consisted of December, January, and February. The diagnoses of all enrolled children were collected using the first three digits of their ICD-9-CM diagnostic codes [32]. The top ten diagnoses from each group were analyzed.

A Microsoft® SQL Server® 2008 R2 was used to retrieve the study sample data from the NHIRD. Microsoft Office Excel 2007 was used for data analysis during this study. SigmaPlot 10.0 (Systat Software Inc. San Jose, CA, USA) was used to create graphical drawings.

## Results

A total of 764,598 children (5% of the whole original data) who required emergency care between 2000 and 2009 were enrolled. Based on the systematic random datasets corrected for the 5% sample, the total case number for all ages who underwent recorded emergency care was 3,056,492 from 2000 to 2009. Thus, children younger than their 18 years old accounted 25% of all individuals requiring emergency care over this period. Among the total enrolled children, 27,948 (3.66%) underwent subsequent hospitalization over the study period. Specifically, from 2004 to 2009, 20,914 children required hospitalization out of 463,264 who sought emergency care, for a calculated average hospitalization rate of 4.51%.

The 5% average annual visiting frequency of children seeking emergency care was 76,460 ± 5,432 visits/year (range: 69,057-86,429), so the estimated whole emergency visit number for children was approximately 1.53 × 10^6^ visits per year. Since the annual population of children younger than 18 years old has declined over the 10 year period from 5.78 to 4.75 million, accounting for approximately 23% of the whole population in Taiwan (Table 1). The annual incidence of emergency visits in children increased from 26,271/100,000 children/year in 2000 to 36,428/100,000 children/year in 2009 (mean ± SD, 29,133 ± 3,104 children /year) (Table 1).

When further analyzing the annual incidence rate of hospitalized or non-hospitalized cases, there was a trend towards a slight increase in the non-hospitalized children (mean ± SD, 28,052 ± 2,838/100,000 children/year). Among the hospitalized children, the annual incidence rate was 630 ± 179/100,000 children/year for 2000 to 2003. However, this might be underestimated. Specifically, the mean value of children requiring hospitalization from the emergency room was 1,381 ± 90/100,000 children/year between 2004 and 2009, which was a more reliable value.

Regarding costs, these were much lower for children compared to adults for both hospitalized and non-hospitalized cases (Table 2). Examining the different age groups of non-hospitalized children, those who were 12-17 years old had the highest cost/visit (NTD $1,511/visit). Nonetheless, the cost/visit did not markedly vary across different age groups for non-hospitalized children. In contrast, for hospitalized children, there was a marked difference among groups. The cost/visit was markedly higher for the admitted 0-11 month-old infant group (NTD$29,160/case) and the admitted 12-17 year-old teenager group (NTD$27,296/case) compared to the 1-5 and 6-11 year-old hospitalized groups (NTD$14,292/case and NTD$17,918/case, respectively) (Table 2).

**Table 2:** Case numbers and expenses of children requiring emergency care (2000-2009)

**Age group**	Case no./year*	Expense/year*	**Cost/case**
	**No. (%)**	**NTD$ (%)**	**NTD$/USD$**
***All ER cases***			
**>17y** (% of all age)	229,189 (75.0)	1.30x10^9^ (89.3)	5,524/183
**0-17y** (% of all age)	76,460 (25.0)	1.55x10^8^ (10.7)	2,032/67
0-5y (% of 0-17y)	43,269 (56.6)	8.81x10^7^ (56.7)	2,056/68
0-11 m (% of 0-17y)	6,919 (9.0)	2.23x10^7^ (14.4)	3,390/112
1-5y (% of 0-17y)	36,350 (47.5)	6.58x10^7^ (42.3)	1,819/60
6-11y (% of 0-17y)	17,904 (23.4)	3.30x10^7^ (21.2)	1,834/61
12-17y (% of 0-17y)	15,288 (20.0)	3.43x10^7^ (22.1)	2,243/74
***ER cases non-hospitalized***			
**>17y** (% of all age)	210,705 (74.1)	4.89x10^8^ (83.3)	2,297/76
**0-17y** (% of all age)	73,666 (25.9)	9.78x10^7^ (16.7)	1,332/44
0-5y (% of 0-17y)	41,363 (56.1)	5.14x10^7^ (52.6)	1,254/41
0-11 m (% of 0-17y)	6,443 (8.8)	7.39x10^6^ (7.6)	1,179/39
1-5y (% of 0-17y)	34,920 (47.4)	4.40x10^7^ (45.0)	1,269/42
6-11y (% of 0-17y)	17,435 (23.7)	2.40x10^7^ (24.5)	1,380/46
12-17y (% of 0-17y)	14,868 (20.2)	2.24x10^7^ (22.9)	1,511/50
***ER cases hospitalized***			
**>17 y** (% of all age)	18,484 (86.9)	8.13x10^8^ (93.4)	41,239/1363
**0-17y** (% of all age)	2,795 (13.1)	5.76x10^7^ (6.6)	19,434/642
0-5y (% of 0-17y)	1,906 (68.2)	3.67x10^7^ (63.8)	18,016/596
0-11 m (% of 0-17y)	476 (17.0)	1.49x10^7^ (25.9)	29,160/964
1-5y (% of 0-17y)	1,430 (51.2)	2.18x10^7^ (37.8)	14,292/472
6-11y (% of 0-17y)	469 (16.8)	8.98x10^6^ (15.6)	17,918/592
12-17y (% of 0-17y)	420 (15.0)	1.19x10^7^ (20.6)	27,296/902

*Data were retrieved and corrected to be 5% of all cases from the random systematic sampling database of Taiwan’s National Health Insurance Research Database.

*Abbreviations:y* years, *m* months, *NTD* new Taiwan dollar, *USD* United States dollar (exchange rate of USD to NTD was 1.00 to 30.25 on February 18, 2014).

**Figure 1:**
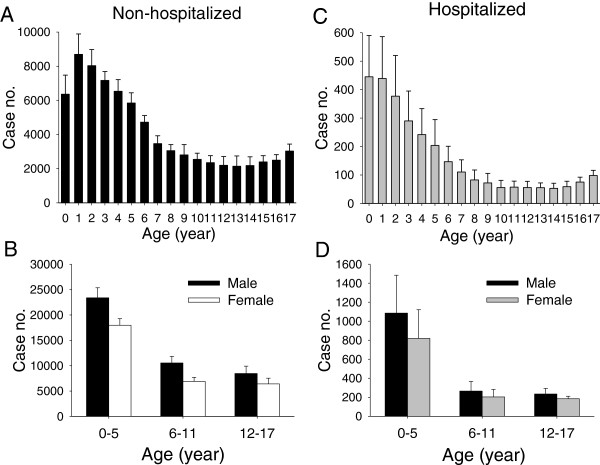
**Age and sex distributions of the children requiring emergency care during the study period (2000-2009).** Data were retrieved, corrected, and presented to be 5% of case numbers per year of all cases from the random systematic sampling database of the National Health Insurance Research Database of Taiwan. **(A)** Age distribution for children not hospitalized after emergency care. **(B)** Different groupings by age and sex of children not hospitalized after emergency care. **(C)** Age distribution of children hospitalized after emergency care. **(D)** Different groupings by age and sex of children hospitalized after emergency care.

The mean age of enrolled children was 6.1 years and in terms of age distribution, there was a decreasing trend of case numbers for children from 1-10 year-old group, followed by a slightly upward trend for the 15-17 year-old group, with or without subsequent hospitalization (Figures 1A and C). When the various groups are summarized, children aged 0-5 years old formed the largest group. In terms of sex, males were more predominant than females in all three age groups regardless of hospitalization (57.5% males vs. 42.5% females) (Figures 1B and D).

**Figure 2:**
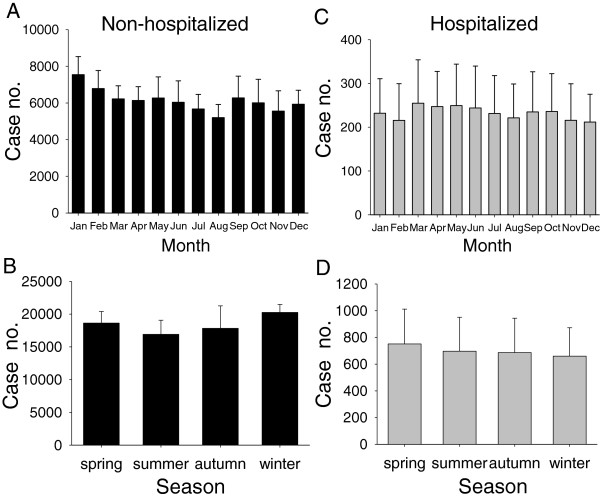
**Monthly and seasonal changes in children requiring emergency visits during the study period (2000-2009).** Data were retrieved, corrected, and presented to be 5% of case numbers per year of all cases from the random systematic sampling database of the National Health Insurance Research Database of Taiwan. **(A)** Monthly and **(B)** seasonal changes of children not hospitalized after emergency care. **(C)** Monthly and **(D)** seasonal changes of children hospitalized after emergency care.

When changes over time and seasonality were examined, emergency visits were highest in winter (20,265 ± 1,215) and lowest in summer (16,918 ± 2,164) for non-hospitalized children (Figures 2A and B). However, for the hospitalized children, there was little difference among seasons, although the highest season was spring (Figures 2C and D).

Among non-hospitalized children, using the first three digits of the ICD-9-CM diagnosis codes, the top ten diagnoses were obtained (Table 3). Specifically, acute airway infection (462,463, 465, and 466), acute gastrointestinal illness (009, 535, 558, 564, 787, and 789), and non-specific general symptoms (780) were the most common diagnoses across all age groups. When individual age groups were examined, it was noted that urinary tract disorders (especially 599.0: urinary tract infection) were common in the 0-11 m infant group, head injuries with an open wound (873) were common among 1-17 year olds, and limb injuries (923, 924) were high in the 12-17 year olds (Table 3).

**Table 3:** Top ten diagnoses of children receiving emergency care without subsequent hospitalization (2000-2009)*

	**0-5 y**		**6-11 y**	**12-17 y**	**0-17y**
**Top**	**0-11 m**	**1-5y**			
1	General symptoms**	AURI	AURI	AURI	AURI
2	AURI	General symptoms	Abdomen/pelvis symptoms	General symptoms	General symptoms
3	Acute pharyngitis	Acute pharyngitis	General symptoms	Abdomen/pelvis symptoms	Acute pharyngitis
4	Acute bronchiolitis	Acute tonsillitis	Gastroenteritis/colitis	Gastroenteritis/colitis	Gastroenteritis/colitis
5	Gastroenteritis/colitis	Gastroenteritis/colitis	Acute pharyngitis	Open wound of head	Abdomen/pelvis symptoms
6	Acute tonsillitis	Acute bronchiolitis	Acute tonsillitis	Acute pharyngitis	Acute tonsillitis
7	GI symptoms	Open wound of head	Open wound of head	Contusion of lower limb	Acute bronchiolitis
8	Urinary tract infection	Abdomen/pelvis symptoms	Acute bronchiolitis	Gastritis/duodenitis	Open wound of head
9	Intestinal infections	GI symptoms	Functional digestive disorders	Acute tonsillitis	Gastritis/duodenitis
10	Abdomen/pelvis symptoms	Gastritis/duodenitis	GI symptoms	Contusion of upper limb	GI symptoms

*Diagnoses were retrieved and sorted using the first three ICD-9-CM codes from the random systematic sampling database of Taiwan’s National Health Insurance Research Database.

**Including non-specific symptoms like fever, convulsion, dizziness, syncope, sleep disorders, malaise and fatigue, et al. (ICD-9-CM: 780).

*Abbreviations:AURI* acute upper respiratory infections, *GI* gastrointestinal system (digestive system), *m* months, *y* years.

Using a similar approach, the top ten diagnoses among hospitalized children for each age group (Table 4) revealed that their disease pattern was very different from that of non-hospitalized children. The diseases of the hospitalized children were more severe and more variable. Their five most common diagnoses were fluid and electrolyte disorders (276), bronchopneumonia (485), gastroenteritis (558), acute bronchitis and bronchiolitis (466), and acute tonsillitis (463), and these were all commonly seen in the 0-11 year old children.

**Table 4:** Top ten diagnoses of children requiring emergency care and subsequent hospitalization (2000-2009)*

	**0-5 y**		**6-11 y**	**12-17 y**	**0-17y**
**Top**	**0-11 m**	**1-5y**			
1	Acute bronchiolitis	Fluid/electrolyte disorder	Fluid/electrolyte disorder	Open wound of head	Fluid/electrolyte disorder
2	Fluid/electrolyte disorder	Bronchopneumonia	Acute tonsillitis	Normal delivery	Bronchopneumonia
3	Gastroenteritis/colitis	Gastroenteritis/colitis	Gastroenteritis/colitis	Acute appendicitis	Gastroenteritis/colitis
4	Urinary tract infection	Acute tonsillitis	Bronchopneumonia	Radius/ulna fracture	Acute bronchiolitis
5	Bronchopneumonia	Acute bronchiolitis	Acute bronchiolitis	Concussion	Acute tonsillitis
6	Bacterial infection	Enterovirus infection	Pneumonia	Fluid/electrolyte disorder	Acute pharyngitis
7	General symptoms	Acute pharyngitis	Acute pharyngitis	Gastroenteritis/colitis	Enterovirus infection
8	Atopic dermatitis	Pneumonia	Asthma	Acute tonsillitis	Pneumonia
9	Enterovirus infection	General symptoms	Acute appendicitis	Contusion of trunk	General symptoms
10	Acute pharyngitis	Otitis media	Gastritis/duodenitis	General symptoms	Urinary tract infection

*The diagnoses were retrieved and sorted using first 3 ICD-9-CM codes from the random systematic sampling database of Taiwan’s National Health Insurance Research Database.

*Abbreviations:m* months, *y* years.

Specifically for the different age groups of hospitalized children, enterovirus infection (074, including 074.0 herpangina and 074.3 hand-foot-mouth disease) among 0-5 year olds; urinary tract infection (599.0) and bacterial infection (041) among infants; acute otitis media (382) among 1-5 year olds; asthma (493) among 6-11 year olds; pneumonia among 1-11 year olds; and acute appendicitis (540) among 6-17 year olds were among the highest diagnoses for each group. Teenagers, the 12-17 year old group, were unique in that they suffered many traumatic injury-related diagnoses, including head injury (873, open wound of head; 850, concussion), upper limb fracture (813), and trunk contusion (922). Moreover, normal delivery (650) was their second most frequent diagnosis (Table 4).

## Discussion

This study demonstrates the 10-year emergency care of children and reveals that the case numbers are higher for younger children and for boys in Taiwan. Acute infectious airway diseases and abdominal illness are the most common diagnoses of all children seeking emergency care. Some children (4.51%) who seek emergency care subsequently require hospitalization for further medical care. The data here can be a reference for future health policy design to improve children’s health care in Taiwan or in other areas of residence of Asian children.

In terms of age, young children aged 0-5 years form the largest group of patients, while infants are the group with the highest hospitalization ratio among all children. Thus, clinicians should pay more attention to little children, especially infants, when they were brought to the emergency room. In addition, the slight increase in the case numbers of children aged 14-17 years old compared to younger children may be due to an increase in their outdoor activities, with a corresponding increase in the risk of traumatic injury.

In terms of NIH cost in Taiwan, children younger than 18 years old only make up approximately 10% of all expenses on emergency care, although children make up 20-25% of the total population of Taiwan and the percentage of emergency visits is 25% for all age groups from 2000-2009. Furthermore, the cost per visit, regardless of hospitalization, is markedly lower for children than for adults (Table 2). A possible reason may be the fact that there is less underlying disease present in children compared to older adults who seek emergency care. Nonetheless, it is also possible that the payment structure for children undergoing NIH care in Taiwan may require a thorough review because there is a unreasonably low payment schedule for children. The medical labor power needed for treating children is usually much higher and the facilities are much more delicate and expensive than those for adults.

There is a markedly higher cost per visit for 0-11 month-old admitted infants and for 12-17 year-old admitted adolescents compared to other age groups. These findings may reflect the more complicated conditions affecting infants and adolescents than children aged 1-11 years old. This implies that medical personnel should pay more attention to children of these particular age groups at the emergency room.

The disease patterns of children requiring emergency care consist of mainly acute illnesses of the respiratory and gastrointestinal systems. Nonetheless, there are variations across different age groups. Based on the analysis, diagnoses of urinary tract infection and acute bronchiolitis are unique to infants, which are also the common reasons for their hospitalization. More severe infectious diseases, including bacterial and enteroviral infections, are also common. These diagnoses form a group of more serious problems among infants, resulting in frequent admission.

Although the disease patterns of 1-5 and 6-11 year-old children are similar, enterovirus infection and acute otitis media among 1-5 year olds and asthma and acute appendicitis among 6-11 year olds are also common reasons for admission. The disease patterns among 12-17 year-old hospitalized children are unique with traumatic injury and pregnancy with childbirth replacing infectious diseases that affect other age groups. Adolescents generally have better immune responses and are therefore less likely to be seriously harmed by common infectious diseases. Instead, they are likely to take part in many more outdoor activities and to start interacting sexually. Such changes seem to results in increased risk of various types of traumatic injuries and in unintended pregnancies.

Acute appendicitis is the third most common diagnosis among 12-17 year-old hospitalized children. This should be taken into consideration among older children who complain of acute abdominal pain. There is a need to pay more attention to their specific problems in order to improve the general health of that specific age group. The specific diseases of different ages may provide useful information for government to design medical policy for children.

Compared to a previous report by Yang et al. that targeted emergency care in general between 2000 and 2004 [27], the present study focuses on children younger than 18 years, includes cases that require subsequent hospitalization, and has a longer study period of 10 years (2000-2009). This study explores the differential disease pattern distributions across different age groups of children and provides a more comprehensive analysis of children seeking for emergency medical help, particularly on how clinicians should pay different attention to children of varying ages.

This study has a number of similarities with the report by Alpern et al. on the pediatric emergency care of children living in United States [33]. These include the mean age (6 years old), the male predominance, and the top two diagnoses (acute upper respiratory infections and fever), even though the ethnicity in Taiwan is almost completely Asian, whereas Asians only account for 1.4% of the population in Alpern study. However, unlike the study by Alpern et al., the present study identifies a slight upward trend in case numbers in the group aged 14-17 years. Furthermore, the hospitalization rate for children is much higher in the United States (11.6%) [33] than in Taiwan (4.51%). A possible explanation is the great convenience and low self-payment ratio of the NHI program in Taiwan. Parents in Taiwan usually do not hesitate to bring their children to the emergency room for help, so cases of non-emergency visits may be higher than that in the United States.

A published report by Tsai et al. analyzes ambulatory visit data in Taiwan for 2002 and demonstrates that approximately 35% of emergency care cases are non-emergency visits or an emergency that is preventable with primary care [28]. After subtracting possible non-emergency cases among the enrolled, the hospitalization rate for real emergency visits in children in Taiwan seems to be about 6.7%, which is still lower than that in the United States. Perhaps associated with this, asthma is the most frequent diagnosed among 5-14 year-old children in the United States, but is only the 8^th^ most frequent diagnosis for 6-11 year-old hospitalized children in the present study (Table 4). Childhood asthma needing emergency care is much less frequent in Taiwan and may be a less serious problem than in the United States.

An important limitation of the present study is the inadequate data on subsequent hospitalization following emergency care for the years 2000 to 2003. This is because the NHI does not strictly require that medical care providers report the hospitalization fee together with the emergency fee during this period. Thus, the hospitalization ratio has been modified to be 4.51% according to the complete admission data for the years 2004 and 2009.

This descriptive analysis presents a detail 10-year nationwide emergency care of children in an Asian island having a well-developed National Health Insurance. The study may provide comprehensive information of Chinese children who require emergency care that may help the health care system make some changes in improving children’s health care, such as education or changes in emergency care policy to improve true emergency care quality and decrease in unnecessary emergency visits. The results may also be a useful reference for Asian children living elsewhere.

## Conclusions

A quarter of all individuals seeking emergency care in Taiwan are children, 4.51% of whom require subsequent hospitalization and further medical care. Young children aged 0-5 years are the largest group. Boys require emergency care more often than girls. The cost per visit and disease patterns varie across different age groups and are especially different for hospitalized infants and 12-17 year-old teenagers. Medical personnel attending all children at the emergency room need to pay attention to different disease patterns based on the children’s age. Preventive measures targeting all children in the areas of respiratory and gastrointestinal diseases, and targeting different diseases of different ages, are important for improving children’s health.

## Abbreviations

NHI: National health insurance; NHIRD: National health insurance research database.

## Competing interests

The authors declared that they have no competing interests. The sponsors had no role in the design, analysis, or presentation of this research.

## Authors’ contributions

MJJ designed the study, analyzed the data, and wrote the manuscript. YSL, PCT, CFY, and WJS were involved in the study design. YCL performed the data analysis and interpretation of the original datasets. All of the authors read and approved the final manuscript.

## Pre-publication history

The pre-publication history for this paper can be accessed here:

http://www.biomedcentral.com/1471-2431/14/100/prepub
